# Latent transition analysis of home-based fluid management during the vulnerable phase in patients with chronic heart failure: impact on symptom burden

**DOI:** 10.3389/fcvm.2026.1811019

**Published:** 2026-06-04

**Authors:** Jing Zhang, Yuanyuan Cai, Xiang Li, Qingyun Song, Xuejiao Sun, Jinmei Yang, Haiyan Yu

**Affiliations:** 1 Department of Cardiac Vascular Surgery, Huai'an Hospital Affiliated to Yangzhou University (The Fifth People's Hospital of Huai'an), Huaian, China; 2 Department of Nursing, Nanjing Luhe People's Hospital, Yangzhou University, Nanjing, China; 3 Department of Cardiovascular Medicine 2, Huai'an Hospital Affiliated to Yangzhou University (The Fifth People's Hospital of Huai'an), Huaian, China; 4 Department of Nursing, Huai'an Hospital Affiliated to Yangzhou University (The Fifth People's Hospital of Huai'an), Huaian, China

**Keywords:** chronic heart failure, home fluid management, latent profile analysis, latent transition analysis, relevance, symptom burden

## Abstract

**Objective:**

To explore the transition in latent categories of home fluid management in patients with chronic heart failure (CHF) over time and their correlation with symptom burden.

**Methods:**

Convenience sampling selected 268 CHF patients. The Home Fluid Management Scale and Memorial Symptom Assessment Scale were utilized to measure the research subjects one month (T1) and three months (T2) after discharge. Latent profile analysis, latent transition analysis, and Logistic regression were applied for statistical testing.

**Results:**

There were 252 valid questionnaires (94.03% effective rate). At T1 and T2, three latent profiles of home fluid management were identified: high, medium, and low-level groups. Latent transition analysis revealed varying stability and transition rates among these groups. For instance, 13.89%, 26.59%, and 9.91% remained persistently in the high, medium, and low-level groups, respectively, while others transitioned between levels (e.g., 7.94% high to medium, 3.17% high to low). Multivariate logistic regression showed that compared to the sustained high-level group, all other groups—including persistent medium (OR=4.477), persistent low (OR=11.917), and all transition groups (e.g., high-medium OR=2.702, high-low OR=6.482)—had significantly increased risks of high symptom burden scores (all *P* < 0.05).

**Conclusions:**

50.39% of the patients with chronic heart failure remained in their original latent state, 21.03% of the patients experienced a deterioration in their management status, and 28.58% of the patients showed positive improvement. Compared with the persistent high - level group, the severity of symptom burden in patients with chronic heart failure, including those in the persistent medium - level group, the persistent low - level group, and various transition groups to the moderate and low - level groups, was significantly higher.

## Introduction

Chronic heart failure (CHF) is the final stage in the development of cardiovascular disease. In the population aged 35 years or older in China, the prevalence is about 1.3%. The number of patients is estimated to be 13.7 million, and it increases significantly with age. In the population aged 70 years or older, the prevalence is more than 10%. The mortality rate is as high as 50%, even higher than that of some malignant tumors in 5 years, imposing a disease burden on society (1, 2).

The vulnerable phase in chronic heart failure (CHF) typically refers to the specific period after hospital discharge for patients admitted with acute decompensated heart failure (ADHF)—generally considered to be within three months post-discharge. During this time, although patients are clinically stabilized and discharged, the risk of cardiovascular death and rehospitalization is significantly elevated, marking a critical transitional period from the acute to the chronic stable phase of the disease (3). Patients in this phase experience unstable cardiac function, with a notably high risk of mortality and readmission. Studies indicate that the readmission rate can reach 25% within 30 days and 40% within 90 days after discharge (4, 5). Proper management of this period can promote and maintain the long - term stability of heart failure symptoms in patients and reduce the occurrence of cardiovascular adverse events. Among them, volume management is the key non - drug management measure (6).

Volume overload is a key factor leading to acute exacerbation and readmission of heart failure. Effective home volume management, including limiting salt intake, controlling fluid intake, and daily monitoring of body weight, is the core of management during this period. It aims to reduce the burden of symptoms such as dyspnea and edema by maintaining fluid balance, thereby reducing the readmission rate (7).

Symptom burden, such as dyspnea, fatigue, and edema, is closely related to volume status (8, 9). Although these symptoms can reveal the association at a certain point in time, they have obvious limitations (9, 10). They cannot reveal the dynamic interaction and causal direction between volume management behavior and symptom burden during the vulnerable phase. They also make it difficult to identify the heterogeneous subgroups and their evolution trajectories in the patient population, and they cannot capture the key turning points affecting prognosis.

This study aims to employ Latent Profile Analysis and Latent Transition Analysis to identify heterogeneous subgroups in home fluid management among patients with chronic heart failure, uncover the dynamic patterns of home fluid management from 1 to 3 months after discharge, and explore the differential impact of transitions in management levels on symptom burden across subgroups. The findings are intended to provide empirical support for developing staged and individualized intervention strategies tailored to patient characteristics, ultimately offering new approaches to reduce symptom burden and improve long-term prognosis.

## Participants and methods

### Participants

From July 2024 to June 2025, we selected 268 patients with chronic heart failure in the Department of Cardiology of the Fifth People's Hospital of Huai'an as subjects by convenience sampling.

Inclusion criteria:

The patients met the diagnostic criteria defined in *the Chinese Guidelines for the Diagnosis and Treatment of Heart Failure 2018* (11).

Hospitalized patients with chronic heart failure.

Basic reading, listening, and speaking skills.

Patients gave informed consent and volunteered to participate in this study.

Exclusion criteria:

Patients with serious diseases such as malignant tumors or other serious comorbidities.

History of mental or cognitive diseases.

They were readmitted to the hospital for other diseases within 3 months.

Death during follow - up or failure to complete 2 longitudinal measurements for other reasons.

This study was conducted after the approval of the hospital ethics committee (202406033). All subjects volunteered to participate in this study and signed the informed consent.

### Research methods

#### Sample size calculation

Sample estimation (12): according to the formula *n* = (*t_α/2_*·*s/δ*)^2^, where *t_α/2_* is the critical value of *α*=0.05 in the t-distribution, *s* is the standard deviation in previous related studies, and *δ* is the allowable error. According to the preliminary experiment, s was 0.79, *δ* was 10%, and *t_α/2_* was 1.96, so *n* = 240 was calculated. Therefore, at least 240 people were needed for this study.

### Survey tools

#### General information questionnaire

The general information questionnaire was designed by the researchers. It includes the patient's age, gender, marital status, education level, occupation, family income, number of comorbidities, left ventricular ejection fraction, and cardiac function classification.

#### Home fluid management self - rating scale

The scale was compiled by You Linbin et al. (13). It includes 4 dimensions: self - care assessment, self - care maintenance, self - care management, and self - care confidence, as well as 27 items. The scale is scored from 1 to 4 points, ranging from “never” to “always”, and the total score ranges from 27 to 108. Evaluation criteria: A score of 27 - 54 points indicates a low degree of individual home fluid management; 55 - 81 points indicates a general degree of individual home fluid management; and 82 - 108 points indicates a high degree of individual home fluid management. The content validity index at the item level ranges from 0.853 to 1.000, and the content validity index at the scale level is 0.951. The total Cronbach's *α* coefficient, split - half reliability coefficient, and test - retest reliability of the scale are 0.930, 0.723, and 0.867, respectively. In this study, the Cronbach's *α* coefficients of the scale at the two measurement time nodes are 0.814 and 0.833.

#### Memorial symptom assessment scale (msas-Hf)

The scale was developed by Zambroski et al. (14) to assess the symptom experience of patients with chronic heart failure in the last 7 days. It includes 3 dimensions, namely physical symptoms, psychological symptoms, and heart - failure symptoms. The patients' symptoms were mainly evaluated from four aspects: the presence or absence of symptoms, the frequency (Likert 1 - 4 score), the severity (Likert 1 - 4 score), and the degree of distress (Likert 0 - 4 score). The mean score of each item represents the score of each symptom burden. Total scores range from 0 to 4, with higher scores indicating a higher level of symptom burden. A score of ≥2 points indicates a high symptom burden (14). The Cronbach's *α* coefficient of the scale was 0.946.

### Data collection methods

Before conducting the survey, all researchers involved in data collection were trained to ensure the standardization of operating procedures. This study strictly adhered to ethical guidelines. The questionnaires were distributed after obtaining hospital permission and informed consent from patients. The patients' general information was extracted from their medical records during their hospital stay. Using a longitudinal design, data from the Home Fluid Management Scale and the Symptom Burden Assessment Scale were collected at 1 month (T1) and 3 months (T2) after hospital discharge. Data were collected through outpatient follow - up, telephone, or WeChat follow - up. To protect patient privacy, all assessments were conducted in a confidential environment, and the questionnaires were anonymized.

### Statistical methods

Latent classes of home fluid management were identified using latent profile analysis, with scores from the four dimensions (self-care appraisal, self-care maintenance, self-care management, and self-care confidence) of the Home Fluid Management Self-Rating Scale included as continuous observed indicators. Model fit was evaluated using the Akaike Information Criterion, Bayesian Information Criterion, sample-adjusted BIC, likelihood-ratio test, and bootstrap likelihood-ratio test, and the number of classes at both time points was determined by comprehensively considering statistical fit indices, class proportions, and clinical interpretability. Latent transition analysis was employed to quantify the dynamic transition probabilities of home fluid management classes between T1 and T2. To further assess the relationship between home fluid management status and symptom burden, multivariable logistic regression was performed with symptom burden scores at T2 as the dependent variable and latent classes of fluid management at T1 and T2 as independent variables. The model was adjusted for baseline characteristics, including age, sex, marital status, education level, occupation, monthly household income, number of comorbidities, left ventricular ejection fraction, and cardiac function class. Prior to modeling, normality tests for continuous variables and homogeneity of variance tests for categorical variables were conducted, and multicollinearity among the included covariates was assessed; all variance inflation factors were below 5, indicating no significant collinearity. The overall goodness-of-fit of the final model was evaluated using the Hosmer–Lemeshow test, and model explanatory power was expressed as Nagelkerke R². All analyses, except for latent profile and transition analyses which were performed in Mplus 8.3, were conducted using SPSS 26.0. A two-tailed *P*-value < 0.05 was considered statistically significant.

## Results

### General information of the respondents

A total of 16 cases lost to follow - up were excluded, and 252 valid questionnaires were collected, with an effective rate of 94.03%. A comparison of baseline characteristics (e.g., age, gender, and educational level) between participants lost to follow-up (*n* = 16) and those who completed the study (*n* = 252) revealed no statistically significant differences (*P* > 0.05). The basic information at T1 is shown in Table 1.

**Table 1:** General information of the subjects (*n* = 252).

**Items**	**Categories**	**Number of cases**	**Percentage (%)**
Age (years)	<45	34	13.49
	45∼59	72	28.57
	≥60	146	57.94
Gender	Male	144	57.14
	Female	108	42.86
Education level	Junior high school or below	169	67.06
	High school	52	20.63
	College and above	31	12.30
Monthly household income (yuan)	<4,000	34	13.49
	4,000 -6,999	88	34.92
	7,000 -9,999	92	36.51
	>10,000	38	15.08
Marital status	Unmarried/divorced/widowed	14	5.56
	Married	238	94.44
Occupation	Enterprises and institutions	44	17.46
	Individual	37	14.68
	Clerk/staff	144	57.14
	No	27	10.71
Number of comorbidities	<5	127	50.40
	≥5	125	49.60
Number of brackets	<2	151	59.92
	≥2	101	40.08
Left ventricular ejection fraction	<40%	37	14.68
	40∼49%	72	28.57
	>50%	143	56.75
Classification of cardiac function	I	28	11.11
	II	85	33.73
	III∼IV	139	55.16

### Systematic bias testing in study design

The common method deviation was tested using Harman's single - factor test. Since this study adopted a longitudinal design, the common method deviation of the data collected from two measurements was examined. The results indicated that the total number of factors with eigenvalues greater than 1 at T1 and T2 were 11 and 10, respectively. The total variance explained by the first common factor was 15.24% and 20.47%, respectively, which was lower than the critical value of 40%. This suggests that there was no serious common method bias in this study.

### Latent profile analysis of home fluid management for patients with chronic heart failure

In this study, the scores from four dimensions of the home fluid management scale were used as assessment indicators. Starting from a baseline model with one latent profile, models containing 1 to 4 latent profiles were sequentially fitted. The results are detailed in Table 2. At both T1 and T2, lower values of AIC, BIC, and aBIC, along with statistically significant LRT and BLRT, indicated better model fit, while an Entropy > 0.8 suggested acceptable classification accuracy. However, the 4-class model showed a non-significant LRT (T1: *P* = 0.105; T2: *P* = 0.124), and one of its latent profiles contained only 5% and 8% of the sample at T1 and T2, respectively, resulting in poor explanatory power. Therefore, the 3-class model was ultimately selected.

**Table 2:** Comparison of fitting parameter indexes of different latent profile models.

Time	Model	AIC	BIC	aBIC	Entropy	LRT	BLRT	Class probability
T1	1	23,740.751	23,765.773	23,742.490				
	2	22,698.864	22,697.785	22,697.283	0.842	0.000	<0.001	0.31/0.69
	3	21,615.362	21,624.289	21,616.296	0.876	0.024	<0.001	0.25/0.47/0.28
	4	20,553.153	20,589.331	20,548.364	0.894	0.105	0.128	0.20/0.15/0.22/0.43
T2	1	23,753.376	23,769.337	23,747.757				
	2	22,627.852	22,628.284	22,633.463	0.864	0.000	<0.001	0.44/0.56
	3	21,536.728	21,548.560	21,538.580	0.892	0.013	<0.001	0.32/0.45/0.23
	4	20,421.276	20,431.564	20,434.707	0.906	0.124	<0.001	0.18/0.29/0.26/0.27

**Figure 1:**
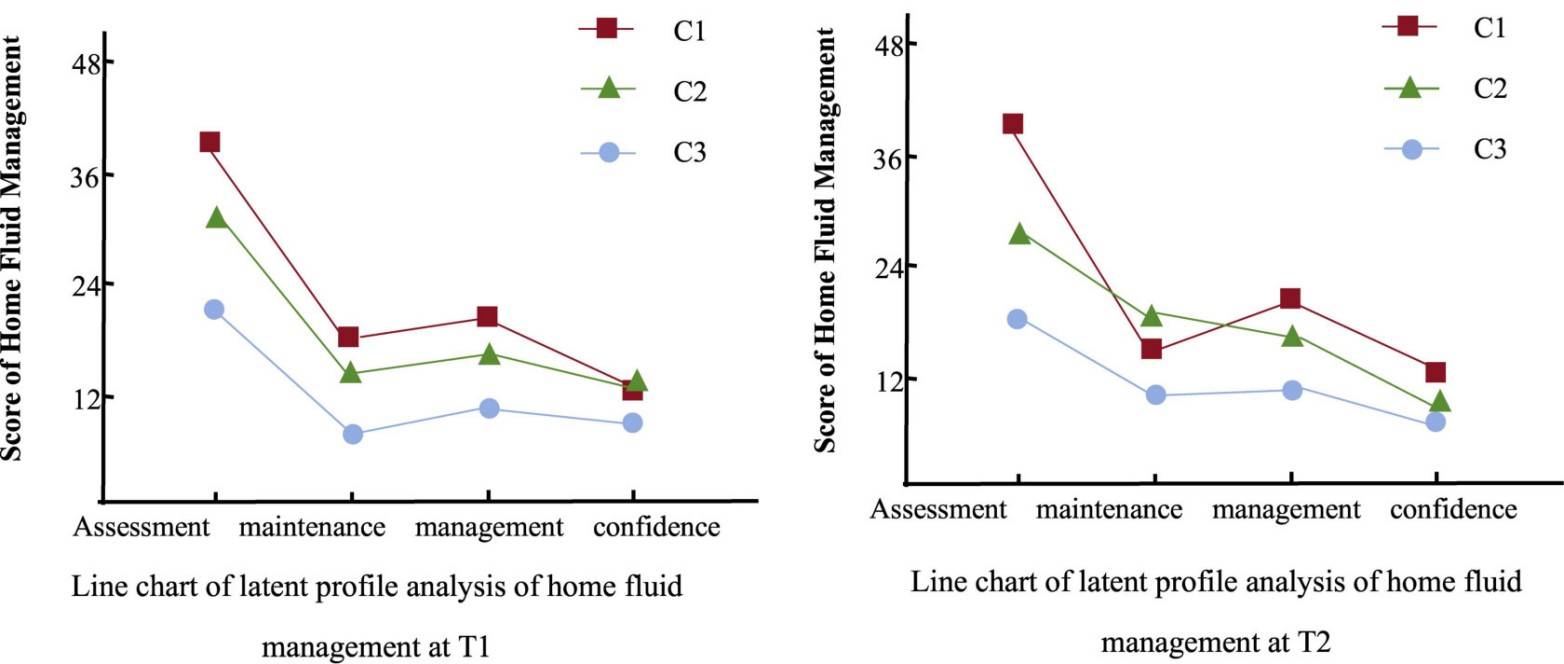
Line chart of latent profile analysis of home fluid management at T1 and T2 in patients with chronic heart failure.

The overall home fluid management score of class 1 (C1) is at a high level, and class 1 is named the high - level group. The overall home fluid management score of class 2 (C2) is at a medium level, and class 2 is named the medium - level group. The overall home fluid management score of class 3 (C3) is at a low level, and class 3 is named the low - level group, as illustrated in Figure 1. The scores of each class (C1, C2, C3) across the four subscales (self-care appraisal, self-care maintenance, self-care management, and self-care confidence) at the T1 and T2 time points are presented in Table 3.

**Table 3:** Scores of the home fluid management self-rating scale for each latent class at different time points (*n* = 252, score, $\bar{x}\pm S$).

Times	Classification	Assessment (6 items)	Maintenance (5 items)	Management (12 items)	Confidence(4 items)
T1	C1	19.89 ± 1.42	18.03 ± 0.68	39.94 ± 2.46	11.88 ± 0.91
	C2	15.52 ± 2.05	13.99 ± 1.02	30.05 ± 3.23	12.92 ± 0.83
	C3	11.07 ± 1.61	8.10 ± 0.97	19.22 ± 2.11	9.11 ± 0.94
T2	C1	21.26 ± 0.82	14.85 ± 1.20	40.01 ± 1.90	12.79 ± 0.89
	C2	15.83 ± 1.95	18.27 ± 0.56	28.13 ± 2.13	11.25 ± 0.80
	C3	12.24 ± 1.80	10.89 ± 0.83	16.87 ± 1.21	9.14 ± 0.89

### Latent transition analysis of home fluid management for patients with chronic heart failure

Latent transition analysis was used to explore the transition of the latent profile of home fluid management in patients with chronic heart failure at T1 and T2 time points. The results showed that 50.39% of patients with chronic heart failure had the probability of maintaining the original latent state. Specifically, 13.89% of chronic heart failure patients remained in the high - level group; therefore, it is named the persistent high - level group. 26.59% of the patients with chronic heart failure continued to be in the medium - level group, so it is named the persistent medium - level group. 9.91% of the patients with chronic heart failure continued to be in the low - level group, so it is named the persistent low - level group. In terms of transitions, 7.94% of the patients with chronic heart failure changed from the high level to the medium level, and 3.17% of the patients changed to the low level. These were named the high - medium transition group and the high - low transition group, respectively. 10.71% of the patients changed from the medium level to the high level, and 9.92% of the patients changed from the medium level to the low level. They were named the medium - high transition group and the medium - low transition group. Also, 7.40% of the patients changed from the low level to the high level, and 10.47% of the patients changed from the low level to the medium level. They were named the low - high transition group and the low - medium transition group, respectively, as shown in Table 4.

**Table 4:** Transition probability of home volume management in patients with chronic heart failure at T1-T2 time points (*n* = 252).

Items		T2		
		C1: High level group	C2: Medium level group	C3: Low level group
T1	C1: High level group	13.89%	7.94%	3.17%
	C2: Medium level group	10.71%	26.59%	9.92%
	C3: Low level group	7.40%	10.47%	9.91%

### Effect of home volume management mode on symptom burden in patients with chronic heart failure

The effect of the transformation mode of home fluid management on the symptom burden of patients with chronic heart failure was further analyzed. Using the transition patterns of home fluid management in chronic heart failure patients as the independent variable and a high symptom burden score (defined as ≥2) as the dependent variable, univariate logistic regression analysis showed that, compared with the persistently high-level group, patients in the persistently medium-level, persistently low-level, high-to-medium transition, high-to-low transition, medium-to-high transition, medium-to-low transition, low-to-high transition, and low-to-medium transition groups all had an increased risk of high symptom burden (*P* < 0.001 for all).

In Model 2 (adjusted for general demographic characteristics such as sex and age) and Model 3 (further adjusted for baseline symptom burden at T1), compared with the persistently high-level group, patients in the persistently medium-level (OR = 4.477), persistently low-level (OR = 11.917), high-to-medium (OR = 2.702), high-to-low (OR = 6.482), medium-to-high (OR = 2.280), medium-to-low (OR = 6.910), low-to-high (OR = 2.664), and low-to-medium (OR = 5.830) groups exhibited an elevated risk of high symptom burden (*P* < 0.05 for all), with the medium-to-high transition group showing the lowest risk (OR = 2.280), as shown in Table 5.

**Table 5:** Association between fluid management at home and symptom burden in patients with chronic heart failure.

Shifting patterns	*B*	*SE*	OR	95%CI	*P*-value
Model 1					
Consistently high level group	-	-	-	-	-
Persistent medium level group	1.651	0.41	5.212	2.334∼11.642	<0.001
Persistently low level group	2.846	0.536	17.219	6.022∼49.233	<0.001
High-medium transition group	1.217	0.33	3.377	1.769∼6.448	<0.001
High-low transition group	2.109	0.492	8.240	3.141∼21.614	<0.001
Medium-high transition group	1.131	0.315	3.099	1.671∼5.745	<0.001
Medium-low transition group	2.235	0.497	9.346	3.529∼24.757	<0.001
Low-high transition group	1.473	0.46	4.362	1.771∼10.747	<0.001
Low-medium transition group	2.014	0.355	7.493	3.737∼15.026	<0.001
Model 2					
Consistently high level group	-	-	-	-	-
Persistent medium level group	1.563	0.379	4.773	2.271∼10.033	<0.001
Persistently low level group	2.647	0.542	11.917	4.878∼40.826	<0.001
High-medium transition group	1.146	0.327	3.146	1.657∼5.971	<0.001
High-low transition group	2.024	0.495	7.569	2.869∼19.969	<0.001
Medium-high transition group	0.925	0.364	2.522	1.236∼5.147	0.015
Medium-low transition group	2.149	0.482	8.576	3.334∼22.059	<0.001
Low-high transition group	1.216	0.416	3.374	1.493∼7.624	0.006
Low-medium transition group	1.836	0.403	6.271	2.847∼13.817	<0.001
Model 3					
Consistently high level group	-	-	-	-	-
Persistent medium level group	1.499	0.391	4.477	2.081∼9.635	<0.001
Persistently low level group	2.478	0.524	11.917	4.267∼33.283	<0.001
High-medium transition group	0.994	0.316	2.702	1.414∼5.020	0.001
High-low transition group	1.869	0.427	6.482	2.807∼14.968	<0.001
Medium-high transition group	0.824	0.328	2.280	1.199∼4.336	0.016
Medium-low transition group	1.933	0.499	6.910	2.599∼18.376	<0.001
Low-high transition group	0.98	0.304	2.664	1.468∼4.835	<0.001
Low-medium transition group	1.763	0.311	5.830	3.169∼10.725	<0.001

Model 1: Logistic regression. Model 2: Adjusted for age, sex, marital status, education level, occupation, household income, number of comorbidities, left ventricular ejection fraction, and cardiac functional class. Model 3: Adjusted for age, sex, marital status, education level, occupation, household income, number of comorbidities, left ventricular ejection fraction, cardiac functional class, and symptom burden at T1. Nagelkerke R² values were 0.18 for Model 1, 0.22 for Model 2, and 0.25 for Model 3. The variance inflation factor for all variables was below 5.

## Discussion

Biegus et al. (15) emphasized that volume management is the core link in the treatment of heart failure, which can effectively reduce the burden on the heart and lower the readmission rate. This study, employing latent profile analysis, identified that home fluid management among patients with chronic heart failure consistently manifested as three statistically stable heterogeneous latent classes (high, medium, and low level) at both 1 month and 3 months after discharge. The prevalence of the low-level group at the two time points was 28.2% and 23.0%, respectively, indicating that even after receiving structured discharge guidance, nearly one-quarter of patients persistently demonstrated significantly inadequate home fluid management capacity during the vulnerable phase. This finding suggests that the current intervention model, predominantly based on universal health education and routine follow-up, has clear limitations in improving patient behavioral adherence, failing to effectively reach and respond to patient subgroups with specific management difficulties (16). To achieve precise intervention for fluid management in the vulnerable phase of chronic heart failure, clinical practice should shift management strategies from a “homogeneous” to a “precision” approach. Specifically, latent profile analysis can be utilized for early identification and continuous monitoring of the low-level management group, systematically assessing specific barriers in dimensions such as self-care appraisal, management behaviors, and confidence. Based on this assessment, tiered interventions can be implemented: for this group, priority should be given to strengthening foundational knowledge and skill training through plain-language education, visual aids, and step-by-step instructional approaches, while also providing structured, individualized follow-up guidance via a continuous support system (e.g., regular follow-ups, remote monitoring, or case management). This model not only helps focus resources and target the reinforcement of patients' fluid management weaknesses but also, through dynamic assessment and early intervention, can effectively reduce the risk of rehospitalization due to inadequate management.

This study, employing latent transition analysis, reveals significant heterogeneity and dynamic evolution in home fluid management status among patients with chronic heart failure. The results indicate that 50.39% of patients maintained their original management status within three months after discharge, suggesting a degree of stability in their disease management trajectory over this period. Conversely, nearly half of the patients (49.61%) underwent transitions in various directions and magnitudes: 21.03% exhibited deterioration in management status, while 28.58% demonstrated improvement. These findings underscore that disease management in chronic heart failure is not static but rather exhibits a distinct bidirectional evolution during the vulnerable phase, wherein a considerable proportion of patients experience a decline in self-management capacity, while another subset achieves behavioral improvement through adaptation and learning. Deterioration in management status may be associated with reduced intensity of medical support after discharge, fluctuations in disease symptoms, diminished self-efficacy, inadequate social support, or the impact of comorbidities. Conversely, improvement may be attributable to effective health guidance, family support, enhanced self-monitoring of symptoms, or strengthened personal health beliefs (17–19). This highlights for clinical practitioners the necessity of recognizing the dynamic nature of patients' management capabilities. Patients should not be viewed as a homogeneous group; instead, early and repeated assessments are essential, with particular attention to those in medium- and low-level groups, as well as those exhibiting negative transitions. Furthermore, interventions should shift from a “one-time” educational approach to a “continuous, stratified, and personalized” support model. For groups with a clear trend toward deterioration, intensified follow-up guidance, symptom alerts, and psychosocial support are warranted, whereas for improving groups, the focus should be on consolidation and positive reinforcement. Finally, healthcare systems can utilize latent profile and transition analyses to identify high-risk populations, optimize resource allocation, and enable precise management and early intervention for patients following different evolutionary trajectories.

The results of this study showed that the severity of symptom burden in patients with chronic heart failure, including those in the persistent medium - level group, the persistent low - level group, and various transition groups to the medium and low - level groups, was significantly higher than that in the persistent high - level group. The results indicate that there is a close negative correlation between the level and dynamic transition of patients' home volume management ability and their clinical symptom experience (20). From the perspective of pathophysiological mechanism, when the patient's volume management remains at a low level or deteriorates, the cardiac pumping function is significantly damaged, and the cardiac output is difficult to meet the basic needs of the body (21, 22). This not only directly leads to hypoperfusion of tissues and organs but also causes symptoms such as fatigue and dizziness, and is more likely to cause fluid retention (23, 24). Fluid retention increases the cardiac preload and the venous pressure of the pulmonary and systemic circulations, which induces or aggravates typical heart - failure symptoms such as dyspnea, cough, and limb edema (25, 26). In addition, poor volume management may reflect excessive activation of the neuroendocrine system, which further accelerates myocardial remodeling and increases peripheral vascular resistance, forming a vicious cycle that increases the symptom burden (27–29).

This suggests that clinical medical staff should go beyond simply focusing on physiological indicators and conduct dynamic monitoring and precise intervention on the overall state of patients, especially their self - management ability and symptom experience. Patients with persistent low or moderate levels or adverse transitions should be considered as high - risk groups for symptom management, and earlier and more active intervention is needed.

Interventions need to be multi - level and individualized, including the intensive use and monitoring of medications such as diuretics to ensure volume balance, as well as individualized fluid and sodium intake programs. Improving patients' health literacy through easy - to - understand health education so that they can accurately recognize symptom transitions and seek help in time is also a key link to alleviate the symptom burden.

This study acknowledges several important limitations. First, the use of a non-probability convenience sample drawn from a single hospital in Huai'an, China, may restrict the generalizability of the transition probabilities and odds-ratio estimates to wider chronic heart failure populations, especially those in rural settings, other geographical regions, or different healthcare systems. Furthermore, both symptom burden and fluid management data were collected via patient self-report, which is susceptible to common-method bias and social-desirability bias; for instance, patients experiencing more severe symptoms may rate their own management more critically. Although Harman's single-factor test was applied to assess common method variance, future research is encouraged to incorporate objective indicators—such as daily weight records and urine-output logs—to allow for multi-dimensional validation. Second, the single-center convenience sampling design limits the representativeness of the sample, and caution is therefore warranted when extending the findings to other settings. Third, although the attrition rate was relatively low (5.97%) and no statistically significant differences in baseline characteristics were observed between study completers and those lost to follow-up, the reasons for attrition—including one mortality—could still influence the comprehensiveness of the results. In addition, measurements were taken at only two time points, which may not adequately capture the nuanced dynamics of self-management behaviors over time. Future investigations should seek to validate these findings through multi-center designs, extended follow-up periods, and the inclusion of objective biomarkers.

## Conclusions

50.39% of patients with chronic heart failure maintained their original latent state, 21.03% of patients experienced a deterioration in their management status, and 28.58% of patients showed positive improvement. Compared with the persistent high - level group, the severity of symptom burden in patients with chronic heart failure, including those in the persistent medium - level group, the persistent low - level group, and various transition groups to the moderate and low - level groups, was significantly higher. The care of patients with chronic heart failure must extend beyond simply focusing on physiological indicators to the dynamic monitoring and precise intervention of the patients' overall state, especially their self - management ability and symptom experience.

## Data Availability

The original contributions presented in the study are included in the article/Supplementary Material, further inquiries can be directed to the corresponding authors.
